# Modulating microRNA expression to produce bigger seeds

**DOI:** 10.1093/plphys/kiad480

**Published:** 2023-09-02

**Authors:** Dechang Cao, Joke De Jaeger-Braet

**Affiliations:** Assistant Features Editor, Plant Physiology, American Society of Plant Biologists; Germplasm Bank of Wild Species, Kunming Institute of Botany, Chinese Academy of Sciences, Kunming, Yunnan 650201, China; Assistant Features Editor, Plant Physiology, American Society of Plant Biologists; Department of Developmental Biology, Institute of Plant Science and Microbiology, University of Hamburg, Hamburg 22609, Germany

To achieve “Zero Hunger,” continuing efforts have been made to increase seed production. Besides seed number, the size and weight of a single seed are also important factors for increasing seed production. Beginning by double fertilization, seed development consists of several distinct phases that involve a combinational effect of maternal and zygotic tissues (Povilus and Gehring 2022). For cereal crops, endosperm takes the majority of the seed volume and is an important source for calories worldwide due to starch production. Upon fertilization, the central cell develops into the endosperm. Extensive nuclei proliferation without cell division occurs in the early endosperm, which results in syncytial endosperm (Fig. 1). Subsequently, cellularization takes place, followed by cell proliferation, cell expansion, and endoreplication, finally developing into mature endosperm (Fig. 1). Both syncytial endosperm proliferation and cell proliferation determine the seed size (Sabelli and Larkins 2009). The newly divided endosperm cells will be filled with starch in later seed development stages, important for nutritional value. Thus, manipulating cell division events in early endosperm development provides an efficient approach to modify seed size (Xu and Zhang 2023). In this issue of *Plant Physiology*, Wang et al. (2023) explored the role of microRNA159 (miR159) in the manipulation of endosperm cell division and grain size.

**Figure 1.**
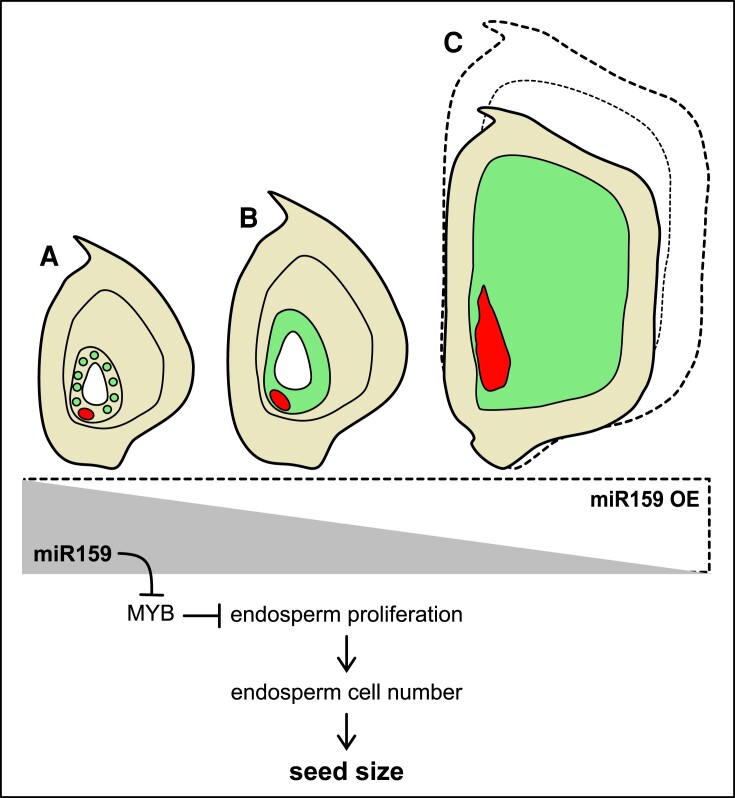
A conceptual model of the role of miR159 during early endosperm development. A simplified representation of postembryonic development from 2 days to 10 days after pollination of endosperm (green) and embryo (red): (a) syncytial endosperm, (b) cellularized endosperm, and (c) mature endosperm [simplified from (Xu and Zhang 2023)]. In maize seeds, miR159 expression gradually decreases during development. MiR159 regulates the size of the endosperm by repressing MYB transcription factors, which in turn repress endosperm proliferation and determine the endosperm cell number and the final seed size. In Zm-miR159-OE maize, seed size was found to be increased by 20% to 30% (dotted lines).

MicroRNAs (miRNAs) are key regulators of gene expression in eukaryotes. MiRNAs have been reported to play multiple roles in regulating seed development, dormancy, and germination (Tognacca and Botto 2021). Wang et al. (2023) profiled small-RNAome of multiple stages during maize (*Zea mays*) seed development and found that *zma-miR159* levels gradually decreased during early seed development. To further investigate the role of *zma-miR159* expression during seed development in maize, the authors overexpressed *zma-miR159* and obtained notably enlarged grains in independent overexpression (*zma-miR159*-OE) lines. The grain weight was increased by around 26% to 30% in the *zma-miR159*-OE lines compared with wild-type plants (Fig. 1). Microscopic observations further revealed an increased endosperm cell number, but no change in cell size in the *zma-miR159*-OE lines, compared with wild-type plants.

In plants, the core sequence of miR159 is highly conserved, and similar isomiRs of miR159 can be found in rice (*Oryza sativa*), Arabidopsis (*Arabidopsis thaliana*), and maize. Considering the conserved sequence of miR159, Wang et al. (2023) overexpressed *zma-miR159* ectopically in rice and Arabidopsis, and similar phenotypes of enlarged seeds were observed. These results suggest that there is a conserved mechanism mediated by miR159 in controlling seed size (Wang et al. 2023).

MiRNAs have been known to regulate gene expression post-transcriptionally by either cleaving the target mRNAs or inhibiting translation of the targets in a sequence-specific manner. For example, zma-miR169o has been shown to negatively regulate *Zm-NF-YA13*, a transcription factor that regulates the expression of *ZmYUC1*, which in turn modulates auxin levels during early seed development (Zhang et al. 2022). Wang et al. (2023) identified targets of miR159 in maize using degradome sequencing. *ZmMYB74*, *ZmMYB138*, *ZmMYB104*, and *ZmMYBr23* were detected to be potential targets of Zma-miR159. Then, 5’ Rapid Amplification of 5’ cDNA Ends was performed to identify *ZmMYB74* and *ZmMYB138* as bona fide targets of miR159. Wang et al. (2023) further validated the regulatory roles of miR159 in regulating the expression levels of *ZmMYB74* and *ZmMYB138*, confirming both being directly targeted by miR159 in maize (Fig. 1).

To validate the function of *ZmMYB74* and *ZmMYB138*, the authors generated knockout lines of *ZmMYB74* using CRISPR/Cas9. Two independent knockout lines showed similar phenotypes to the *miR159*-OE lines, with increased seed size and weight (Wang et al. 2023). Although more detailed microscopic observations of endosperm cells in the *ZmMYB74* knockout lines were not shown, these results suggest that the negative regulation of *ZmMYB74* by miR159 leads to enlarged seed size (Fig. 1).

MYBs are transcription factors involved in various aspects of plant growth and development. The close homologues of *ZmMYB74* and *ZmMYB138* in Arabidopsis are *GAMYB33* and *GAMYB101*, which have also been reported as targets of miR159 (Reyes and Chua 2007). It was revealed, using reverse transcription quantitative PCR, that *ZmMYB74* and *ZmMYB138* were expressed in various tissues, showing the highest expression levels in seeds at 3 days after pollination, after which the expression rapidly decreased. This expression analysis clearly suggests their role in early endosperm development (Wang et al. 2023).

To further explore how ZmMYB74 and ZmMYB138 are involved in seed size regulation, Wang et al. (2023) performed DNA affinity purification sequencing (DAP-seq) to identify their downstream genes. Binding sites of these transcription factors could be detected on all the maize chromosomes, being concentrated at 2 kb upstream of the transcription start sites and 1 kb downstream of the transcription termination sites. Around 18% of the ZmMYB-binding peaks were identified in promoter regions, which revealed 2,507 and 5,096 putative target genes of ZmMYB74 and ZmMYB138, respectively (Wang et al. 2023). Most interestingly, the promoter region of cyclin dependent kinase (CDK) was among the downstream targets. CDK is one of the major regulators of cell division, and its expression was increased in *Zm-miR159-*OE lines (Wang et al. 2023).

The final size of a plant organ, including grains, is determined by tight coordination of cell proliferation and cell expansion during development (Sabelli and Larkins 2009; Xu and Zhang 2023). For agricultural purpose, it is of great interest to fully unravel the regulatory mechanisms and find ways to overcome the size constrains. The research by Wang et al. (2023) shows that the miR159-MYB74/138 pathway has potential in increasing grain size. More research is needed to realize the potential of miR159 in crop production. For example, miR159 has also been reported to be involved in regulation of seed dormancy of *Cunninghamia lanceolata* (Cao et al. 2016). It is very likely that miR159 affects other developmental processes, because it targets mRNAs of abscisic acid–associated genes (Reyes and Chua 2007). Lastly, it is common to have trade-offs between desired agricultural traits. Therefore, there could be, for example, a compromise between endosperm cell number and starch accumulation. It might be necessary to evaluate the starch content in *Zm-miR159*-OE lines to address this possibility.

## Dive Curated Terms

The following phenotypic, genotypic, and functional terms are of significance to the work described in this paper:
